# Isolation and Characterization of Facultative-Anaerobic Antimonate-Reducing Bacteria

**DOI:** 10.3390/microorganisms8091435

**Published:** 2020-09-18

**Authors:** Ziran Yang, Hisaaki Hosokawa, Takuya Sadakane, Masashi Kuroda, Daisuke Inoue, Hiroshi Nishikawa, Michihiko Ike

**Affiliations:** 1 Division of Sustainable Energy and Environmental Engineering, Graduate School of Engineering, Osaka University, 2-1 Yamadaoka, Suita, Osaka 565-0871, Japan; yang@wb.see.eng.osaka-u.ac.jp (Z.Y.); hosokawa@wb.see.eng.osaka-u.ac.jp (H.H.); sadakane@wb.see.eng.osaka-u.ac.jp (T.S.); kuroda@sz.tokoha-u.ac.jp (M.K.); d.inoue@see.eng.osaka-u.ac.jp (D.I.); 2 Faculty of Social and Environmental Studies, Tokoha University, 6-1 Yayoi-cho, Suruga-ku, Shizuoka, Shizuoka 422-8581, Japan; 3 Joining and Welding Research Institute, Osaka University, 11-1 Mihogaoka, Ibaraki, Osaka 567-0047, Japan; nisikawa@jwri.osaka-u.ac.jp

**Keywords:** antimonate-reducing bacteria, antimony, *Dechloromonas* sp., facultative anaerobe, *Propionivibrio* sp.

## Abstract

Microbial antimonate (Sb(V)) reduction is a promising approach to remove Sb(V) from wastewater. However, current knowledge regarding microbial Sb(V) reduction is limited to strictly anaerobic conditions. This study was the first to isolate three facultative-anaerobic Sb(V)-reducing bacterial strains from the sludge collected from a wastewater treatment facility in an antimony products plant. Two of the isolated strains, designated *Dechloromonas* sp. AR-2 and *Propionivibrio* sp. AR-3, were characterized based on their Sb(V)-reducing abilities. When cultivated under anaerobic conditions with Sb(V) and acetate as the electron acceptor and donor, respectively, both strains could efficiently reduce 5.0 mM Sb(V), removing most of it from the water phase within 7 d. Along with Sb(V) reduction by the strains, white precipitates, which were likely amorphous Sb(OH)_3_ solids, were formed with a minor generation of soluble antimonite. Additionally, respiratory Sb(V) reduction by both strains occurred not only under anaerobic but also microaerobic conditions. It was suggested that Sb(V) reduction and the growth abilities of the strains under microaerobic conditions presented a substantial advantage of the use of strains AR-2 and AR-3 for practical applications to Sb(V)-containing wastewater treatment.

## 1. Introduction

The metalloid antimony (Sb) is a type of trace element that is widely utilized as a semiconductor material, catalyst for polyethylene terephthalate synthesis, flame retardant, and brake lining [1]. Although Sb can exist in four oxidation states (-III, 0, III, and V), in nature it is usually encountered in the III or V states [2], and Sb(OH)_3_ (antimonite) and Sb(OH)_6_^−^ (antimonate) are the most common Sb compounds in the aquatic environment [3,4].

As the ninth most mined metal worldwide [5], Sb is mainly released in the environment by mining and smelting processes [6]. Consequently, Sb pollution has been detected in various aquatic environments [7,8,9,10]. Because Sb and its compounds are highly toxic chemicals and even suspected carcinogens, they have been classified as pollutants of priority interest by the US Environmental Protection Agency and the European Union [1,6]. Therefore, the development of an Sb-removal method is highly desired to avoid potential human risks from Sb pollution.

Several physicochemical methods such as adsorption, coagulation, membrane separation, electrochemical treatment, ion exchange, and extraction have been applied in Sb removal [1,11]. However, these methods have weaknesses, such as high energy and cost requirements, generation of a large amount of by-products (e.g., adsorbents used for adsorption treatment and inorganic sludge generated by coagulation), and dependence of removal efficiency on the chemical form of Sb [11].

A biological method that applies heterotrophic anaerobic respiration by dissimilatory Sb(V)-reducing bacteria has been recently proposed as an alternative approach for the removal of Sb from the aqueous phase (i.e., wastewater) [12]. The existence of dissimilatory Sb(V)-reducing bacteria, which are capable of utilizing Sb(V) as the electron acceptor in heterotrophic anaerobic respiration, in the environment has been demonstrated by microcosm studies [13,14,15]. Dissimilatory reduction of Sb(V) to Sb(III) enables Sb removal from the aqueous phase because of the insoluble characteristics of Sb(III) compounds. To date, two different types of dissimilatory Sb(V)-reducing bacteria, *Desulfuribacillus stibiiarsenatis* MLFW-2 and *Sinorhizobium* sp. JUK-1 [16,17], have been isolated and characterized for their Sb(V) reduction abilities. It was found that Sb(V) reduction by these strains generated insoluble Sb_2_O_3_ and Sb(OH)_3_ precipitates, respectively. Thus, these studies have shown the feasibility of Sb(V) bioreduction for aqueous Sb removal. However, both strains are obligate anaerobes and require strictly anaerobic conditions for cultivation, thereby limiting the range of their application. To our knowledge, no Sb(V)-reducing strain capable of growing under aerobic conditions or even reducing Sb(V) in the presence of oxygen has been found, despite the usefulness of such a strain in practical Sb-removal technology.

In this study, Sb(V)-reducing bacterial strains capable of aerobic growth were successfully isolated from sludge collected from a wastewater treatment facility in an antimony products plant. The isolated strains were phylogenetically identified and their growth and Sb(V) reduction capability were characterized, including the effects of aerobic conditions. The precipitates formed by Sb(V) reduction by the isolated strains were also characterized.

## 2. Materials and Methods

### 2.1. Culture Media and Cultivation Conditions

Anoxic minimal medium (AMM) [18] was used to enrich, cultivate, and characterize Sb(V)-reducing bacteria. AMM was prepared with ultrapure water and contained the following ingredients: NaCl 1.2 g/L, KCl 0.3 g/L, NH_4_Cl 0.3 g/L, KH_2_PO_4_ 0.2 g/L, Na_2_SO_4_ 0.009 g/L, MgCl_2_·6H_2_O 0.4 g/L, CaCl_2_·2H_2_O 0.15 g/L, H_3_BO_3_ 0.06 mg/L, CoCl_2_·6H_2_O 0.19 mg/L, CuCl_2_·6H_2_O 0.002 mg/L, MnCl_2_·4H_2_O 0.1 mg/L, ZnCl_2_ 0.07 mg/L, FeCl_2_·4H_2_O 1.5 mg/L, NaMoO_4_·6H_2_O 0.036 mg/L, NiCl_2_·6H_2_O 0.024 mg/L, 25% HCl 0.01 mL/L, vitamin solution 10 mL/L and HEPES 20 mM. Vitamin solution containing *p*-aminobenzoic acid 5 mg/L, biotin 5 mg/L, folic acid 2 mg/L, lipoic acid 5 mg/L pyridoxine-HCl 1 mg/L, riboflavin-HCl 5 mg/L, niacinamide 5 mg/L, D-Ca-pantothenate 5 mg/L and cyanocobalamin (B_12_) 0.1 mg/L [19]. K[Sb(OH)_6_] was added at 5 mM as the source of Sb(V) to AMM. Sodium lactate or sodium acetate was added at 5 mM as the sole carbon source to AMM containing Sb(V), and these media were designated L-Sb(V)-AMM and A-Sb(V)-AMM, respectively. The pH of both media was adjusted to 7.0. To isolate and cultivate Sb(V)-reducing bacteria, 1/10 strength Tryptic Soy Broth (1/10 TSB; Becton, Dickinson and Company, Sparks, USA) and LB broth (Lennox; Becton, Dickinson, and Company, Sparks, USA) were also used. Agar was added at 1.5% (w/v) to make solid media. Unless otherwise noted, cultivation was conducted at 28 °C. For aerobic and microaerobic cultivation, the liquid culture was sealed with a silicone sponge plug; then, the culture was incubated with rotary shaking at 120 rpm for aerobic conditions, whereas it was incubated statically without shaking for microaerobic conditions. The creation of microaerobic condition with static cultivation has been verified previously [20]. Anaerobic cultivation of the liquid culture was conducted with rotary shaking at 120 rpm after being sealed with a butyl rubber septum and an aluminum crimp and purged with nitrogen gas for 15 min.

### 2.2. Enrichment of the Sb(V)-Reducing Bacteria

The sludge used as the inoculum for the enrichment of Sb(V)-reducing bacteria was collected from a sedimentation basin in the wastewater treatment facility in an antimony product plant in Hyogo, Japan. The sample was transported on ice to the laboratory and stored at 4 °C until use. The pH of the sludge sample was 7.3 and its aqueous phase contained Sb at 1.95 mg/L.

Before enrichment of the Sb(V)-reducing bacteria, the sludge sample (5 g-wet) was added to 50 mL of L-Sb(V)-AMM, excluding Sb(V), in a 100 mL glass vial. After creating anaerobic conditions as described above, the culture was shaken at 120 rpm and 28 °C for 30 min and homogenized using a VCX130PB ultrasonic processor (Sonics and Materials, Newtown, USA) for 1 min to disperse the flocs. Four milliliters of the pretreated inoculum culture was inoculated into 36 mL of L-Sb(V)-AMM in a 100 mL glass vial. Then, the culture was incubated anaerobically. Aliquots (4 mL) of the culture were subcultured in 36 mL fresh medium at an interval of 10 d from the 1st to 4th batch cycles and 7 d after the 5th batch cycle.

### 2.3. Isolation of Sb(V)-Reducing Bacteria

The enrichment culture after the 6th batch cycle was spread on 1/10 TSB and L-Sb(V)-AMM agar media and cultivated at 28 °C under aerobic and anaerobic conditions, respectively. Morphologically different colonies appearing on each medium were repeatedly transferred to isolate Sb(V)-reducing strains.

### 2.4. Phylogenetic, Physiological and Biochemical Characterization

The isolated Sb(V)-reducing strains were phylogenetically identified based on partial 16S rRNA gene sequences. Genomic DNA was extracted using the Cica Geneus DNA extraction reagent (Kanto Chemical, Tokyo, Japan). Partial sequences of the 16S rRNA genes were amplified using universal primers 27F and 1392R [21,22]. The PCR products were purified using a NucleoSpin Gel and PCR Clean-up kit (Takara Bio, Kusatsu, Japan), and sequenced by Macrogen Japan (Kyoto, Japan). The obtained sequences were compared with sequences in the NCBI database using the BLAST search program (http://www.ncbi.gov/blast/). The partial 16S rRNA gene sequences of isolated strains were deposited in GenBank/EMBL/DDBJ databases under the accession numbers LC569773 to LC569775.

The physiological and biochemical characterization of isolated strains were entrusted to Techno Suruga Laboratory (Shizuoka, Japan). Microscopic observations of cell morphology, and catalase, oxidase, oxidation-fermentation (O/F), and organic compounds utilization tests were conducted.

### 2.5. Characterization of Growth Ability

The isolated strains were precultured in LB broth at 28 °C under aerobic conditions for 24–48 h. The cells were collected by centrifugation (6000× *g*, 4 °C, 10 min), inoculated into fresh LB broth, and cultured again for 7 h. Thereafter, the cells were collected in the same manner, washed with an inorganic solution (Table S1), and inoculated into 5 mL of LB broth in L-shaped test tubes to yield an optical density of 0.05 at 600 nm (OD_600_). The effects of temperature on the growth of isolated strains were tested in LB broth (pH 7.8) at 20, 28, 37, and 45 °C. The effects of pH were examined at 28 °C using LB broth whose initial pH was adjusted to 5.0–8.5 using the appropriate buffer (Table S2). All cultivations used to examine the effects of temperature and pH on growth were conducted in a TVS062CA compact rocking incubator (Advantec, Tokyo, Japan) with shaking at 70 rpm, and the optical density of the culture at 660 nm was automatically recorded every 30 min. All experiments were run in triplicate.

### 2.6. Sb(V) Reduction Experiments

To characterize the ability of Sb(V) reduction, the isolated strains were precultivated as described above and inoculated into 20 mL of A-Sb(V)-AMM in a 50 mL glass vial at an initial OD_600_ of 0.05. The culture was incubated under aerobic, microaerobic, and anaerobic conditions. Controls without inoculation of the isolated strains or supplementation with acetate were also prepared. Multiple identical cultures for the test and control systems were prepared, and three vials were sacrificed temporally to monitor variations in Sb, acetate, and protein concentrations during the Sb(V) reduction experiments. Because the formation of precipitates might disturb the determination of OD_600_, protein concentration, which has been confirmed to be positively correlated to cell concentration (Figure S1), was used to represent the concentration of isolated strains during Sb(V) reduction experiments.

### 2.7. Analytical Methods 

Before analyses of soluble Sb and acetate, the collected samples were filtered through a 0.2 μm cellulose acetate membrane filter (Advantec). Inductively coupled plasma-atomic emission spectrometry (SPS7800, SII Nano Technology, Tokyo, Japan) was used to determine the concentration of total soluble Sb. The speciation of Sb(V) and Sb(III) was performed using a Shimadzu LC-20A high-performance liquid chromatography system (HPLC; Shimadzu, Kyoto, Japan) coupled with hydride generation-atomic fluorescence spectrometry (HG Millennium Excalibur System; P S Analytical, Orpington, UK). A PRP-X100 anion-exchange HPLC column (Hamilton, Reno, USA) and a mobile phase of 200 mM ammonium tartrate (pH 5.0) were used for the separation of Sb(V) and Sb(III), and the separated Sb species were detected with a P802SF hollow cathode lamp for Sb (Photron Pty, Narre Warren, Australia). The acetate concentration was determined with a Shimadzu LC-10A HPLC system (Shimadzu) equipped with an Aminex HPX-87H ion exclusion column (Bio-Rad Laboratories, Hercules, California, USA) using a mobile phase of 5.0 mM sulfuric acid.

The OD_600_ of the culture was measured with a UV-1850 UV–vis spectrophotometer (Shimadzu). The protein concentration was determined using a BCA Protein Assay kit (Takara Bio) and an Epoch 2 microplate spectrophotometer (BioTek, Winooski, Vermont, USA).

### 2.8. Solid Analysis

For analysis of precipitates, the washed cells were inoculated into 60 mL of A-Sb(V)-AMM in a 100 mL glass vial at an initial OD_600_ of 0.05 and cultivated anaerobically for 7 or 35 d. The culture was centrifuged (2000× *g*, 24 °C, 1 min), and the recovered precipitates were rinsed successively with ultrapure water, 100% acetone, and ultrapure water. After removing the supernatant, the precipitates were vacuum-dried and stored in a desiccator until use.

The morphology and elemental distributions of the solids were analyzed through scanning electron microscopy coupled with energy-dispersive X-ray spectroscopy (SEM–EDX; SU-70, Hitachi, Tokyo, Japan) at accelerating voltages of 10 or 15 kV. The crystal structure was analyzed by X-ray diffraction (XRD) using a Rigaku Ultima IV high-performance, multipurpose XRD system (Rigaku, Tokyo, Japan) at 40 kV and 40 mA.

## 3. Results and Discussion

### 3.1. Isolation and Identification of Sb(V)-Reducing Bacteria

Enrichment of Sb(V)-reducing bacteria from the inoculum sludge was conducted with six sequential batch cultivations. In the first batch of the enrichment experiment, soluble Sb was removed sufficiently, and yellowish precipitates were formed in the culture (Figure S2). Thereafter, although Sb removal occurred stably, the color of precipitates gradually changed during the enrichment process and became white by the sixth batch (Figure S2). According to the evidence provided in previous studies, the white precipitates were likely Sb_2_O_3_ formed by microbial Sb(V) reduction [16]. These results indicated the enrichment of Sb(V)-reducing bacteria during the enrichment process.

Isolation of Sb(V)-reducing bacteria from the enrichment culture was attempted after the sixth batch with two distinct cultivation conditions. Consequently, two strains, designated strains AR-1 and AR-2, and one strain, designated strain AR-3, were isolated successfully by aerobic cultivation on 1/10 TSB agar medium and anaerobic cultivation on L-Sb(V)-AMM agar medium, respectively. Based on the partial 16S rRNA gene sequences, all three strains belonged to the order *Rhodocyclales* of the class β-*Proteobacteria*. The sequences of strains AR-1 and AR-3 (1292 and 1294 bp, respectively) shared identical sequences for 1292 bp (an additional nucleotide at both the 5ʹ- and 3ʹ-ends occurred in the sequence for strain AR-3) and had 99.7% and 99.8% nucleotide similarities with that of *Propionivibrio militaris* MP^T^ (accession No. NR_125528), respectively (Table 1). The 16S rRNA gene sequence of strain AR-2 (1290 bp) had 98.4% nucleotide similarity with that of *Dechloromonas agitata* CKB^T^ (accession No. NR_024884). Thus, the isolated strains were identified as *Propionivibrio* sp. AR-1, *Dechloromonas* sp. AR-2, and *Propionivibrio* sp. AR-3. Because of the identical 16S rRNA gene sequences, strain AR-3 was chosen over strains AR-1 and AR-3 as the representative for further characterization.

### 3.2. Physiological and Biochemical Characteristics of Strains AR-2 and AR-3

Both of strains AR-2 and AR-3 were Gram-negative, rod-shaped, non-spore-forming motile bacteria, and positive for catalase, oxidase, nitrate reduction, and cytochrome oxidase tests (Table 1, Table S3). Notably, both strains were capable of growing under both aerobic and anaerobic conditions. Additionally, they can grow in LB medium under the following temperature and initial pH conditions: 15–37 °C and pH 6–8.5 for strain AR-2 and 15–37 °C and pH 5–8.5 for strain AR-3. These fundamental physiological and biochemical characteristics of strains AR-2 and AR-3 were similar to those of *D. agitata* and *P. militaris* [23,24], respectively. However, neither strains AR-2 nor AR-3 could assimilate most of the carbohydrates and fatty acids tested, including glucose and citrate, which required further study to explore the preferable substrates for their growth.

### 3.3. Anaerobic Sb(V) Reduction by Strains AR-2 and AR-3

**Figure 1:**
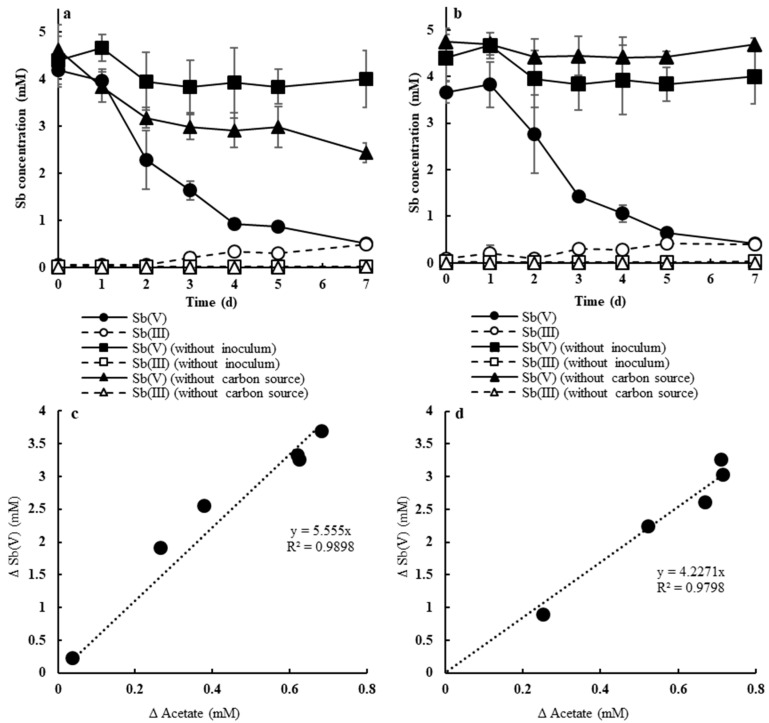
Time course of soluble Sb(V) and Sb(III) (**a**,**b**) and the relationship between acetate consumption (ΔAcetate) and Sb(V) decline (ΔSb(V)) (**c**,**d**) during anaerobic Sb(V) reduction by strains AR-2 (**a**,**c**, respectively) and AR-3 (**b**,**d**, respectively). Error bars represent the standard deviation (*n* = 3).

Time courses of soluble Sb(V) and Sb(III) concentrations during anaerobic Sb reduction experiments are shown in Figure 1. No significant Sb(V) reduction occurred in the abiotic control. When strains AR-2 and AR-3 were inoculated with the addition of acetate, the Sb(V) concentration in the liquid phase declined concomitantly with acetate consumption after 2 d and reached 0.4–0.5 mM after 7 d (Figure 1a,b and Figure S3).

Along with the decline of soluble Sb(V), Sb(III) was detected in the liquid phase and reached 0.4–0.5 mM after 7 d. Additionally, white precipitates were observed during the Sb(V) reduction (Figure S4). These results indicate that both strains AR-2 and AR-3 were capable of respiratory Sb(V) reduction, and a large portion of Sb(III) formed by Sb(V) reduction was removed from the liquid phase as insoluble Sb compounds. Among the two previously isolated Sb(V)-reducing bacterial strains, *D. stibiiarsenatis* MLFW-2 could completely reduce 2 mM Sb(V) within approximately 80 h [16]. Another Sb(V)-reducing strain, *Sinorhizobium* sp. JUK-1, reduced half of 5 mM Sb(V) after 100 h, and further reduced it to less than 0.5 mM after approximately 150 h, with the remaining soluble Sb(III) being 3 mM [17]. Thus, the Sb(V) reduction and removal abilities of strains AR-2 and AR-3 were comparable to or higher than those of previous isolates.

Anaerobic respiration using Sb(V) and acetate as the electron acceptor and donor, respectively, can be described with the following equation [17]:

4Sb(OH)_6_^−^ + CH_3_COO^−^ + 3H^+^ → 4Sb(OH)_3_ + 2HCO_3_^−^ + 8H_2_O

According to the equation, the molar ratio of Sb(V) reduction (∆Sb(V)) to acetate consumption (∆Acetate) during anaerobic respiration should be 4:1. As shown in Figure 1c,d, linear correlations between ∆Sb(V) and ∆Acetate occurred during Sb(V) reduction with both strains. The cell growth of both strains was also correlated with ∆Sb(V) and ∆Acetate (Figure S5), which suggests the strains can obtain energy for proliferation from the reactions. The ratio of ∆Sb(V) to ∆Acetate during Sb(V) reduction by strain AR-2 was 4.2:1, which was close to the theoretical value. On the other hand, the ∆Sb(V)/∆Acetate ratio during Sb(V) reduction by strain AR-2 was 5.6:1, which was higher than the theoretical value. Although Sb(V) reduction by strain AR-3 did not occur without the addition of acetate (Figure 1b), the slight Sb(V) reduction by strain AR-2 was observed even when acetate was not added (Figure 1a). *Dechloromonas* spp., to which strain AR-2 belonged, has been known to accumulate polyhydroxyalkanoates (PHAs) as intracellular carbon and energy storage compounds [25]. Thus, larger Sb(V) reduction by strain AR-2 than that estimated theoretically from acetate consumption was probably caused by utilizing PHA as an additional electron donor for anaerobic Sb(V) respiration.

### 3.4. Effects of Oxygen on Sb(V) Reduction by Strains AR-2 and AR-3

**Figure 2:**
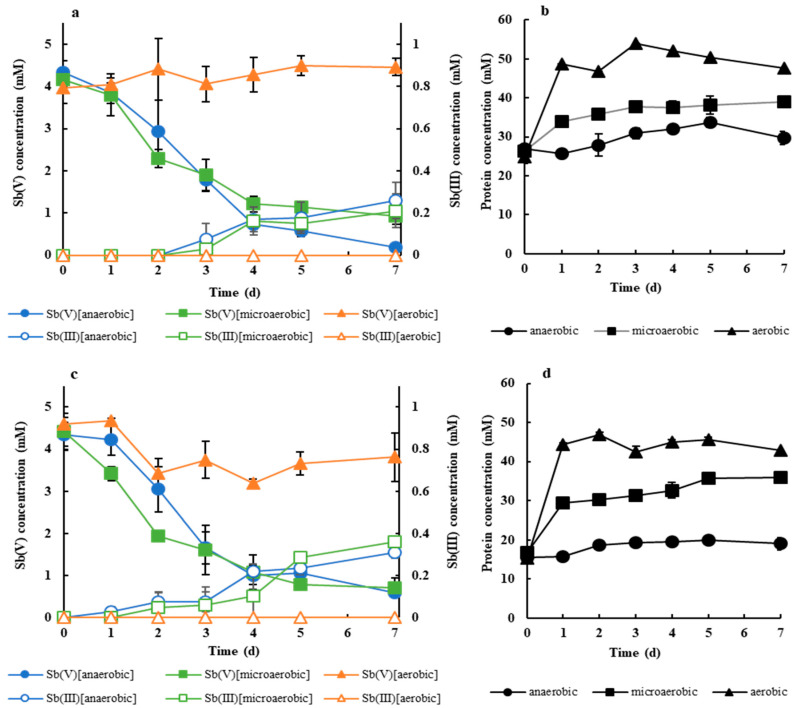
Time course of soluble Sb(V) and Sb(III) (**a**,**c**) and protein concentrations (**b**,**d**) during Sb(V) reduction experiments under anaerobic, microaerobic, and aerobic conditions by strains AR-2 (**a**,**b**, respectively) and AR-3 (**c**,**d**, respectively). Error bars represent the standard deviation (*n* = 3).

In contrast to Sb(V)-reducing bacteria isolated in previous studies, which were obligate anaerobes [16,17], both stains AR-2 and AR-3 were facultative anaerobes. Therefore, the possibility of Sb(V) reduction by the strains not only under the anaerobic conditions but also under aerobic or microaerobic conditions was investigated. During the Sb(V) reduction experiment using strain AR-2 under aerobic conditions, Sb(V) reduction was not observed, although the cell density (protein concentration) increased sharply concomitant with the rapid consumption of acetate (Figure 2a,b and Figure S6). When strain AR-3 was used, rapid growth with acetate consumption was also found and the Sb(V) concentration declined marginally from 1 to 2 d; however, Sb(III) was never detected (Figure 2c) and the total soluble Sb concentration remained unchanged (data not shown). Therefore, it is suggested that Sb(V) reduction by both strains is not possible under aerobic conditions. In contrast, obvious Sb(V) reduction by both strains was detected under the microaerobic conditions with the appearance of Sb(III) and relatively rapid cell growth (Figure 2 and Figure S6). Notably, Sb(V) reduction efficiency by strain AR-3 under microaerobic conditions was comparable to that under anaerobic conditions (Figure 2c). Sb(V) reduction by strain AR-2 under microaerobic conditions was also similar to that under anaerobic conditions during the initial period, although it slowed during the latter period (Figure 2a). These results indicated that both strains AR-2 and AR-3 were capable of Sb(V) reduction even under microaerobic conditions (i.e., in the partial presence of dissolved oxygen). It was also worth noting that both strains were capable of growing more efficiently under microaerobic conditions than under anaerobic conditions even when Sb(V) was used as the electron acceptor.

To our knowledge, this was the first study indicating the possibility of microbial Sb(V) reduction without strict control of anaerobic conditions by pure strains. Under microaerobic conditions, dissolved oxygen in the culture was consumed through the initial rapid growth of strains AR-2 and AR-3, which enabled the occurrence of their anaerobic respiration using Sb(V) as the electron acceptor. The Sb(V) reduction and growth abilities of the strains under microaerobic conditions present a great advantage for strains AR-2 and AR-3 in practical applications for Sb(V) treatment.

Sb(V) bioreduction by biofilms have been reported in recent studies [26,27]. Of these, a study has reported the occurrence of Sb(V) bioreduction even in the presence of oxygen at ~0.2 to 1.8 mg/L in the influent by methane-fed biofilms, and suggested that *Thermomonas* was responsible for Sb(V) reduction by 16S rRNA gene amplicon sequencing [26]. This study also found that a strong aerobic condition (oxygen concentration: 8 mg/L) impaired Sb(V) reduction by the biofilm along with the decrease in the *Theromonas* population, because *Thermomonas* exist usually under anaerobic conditions. However, the findings in this study suggest that biofilm formation might mitigate the detrimental effects of oxygen on Sb(V) reduction and be advantageous for Sb(V) reduction under slightly aerobic conditions. Thus, the biofilm formation ability of the isolated strains and the relationship, if any, between biofilm formation and Sb(V) reduction abilities are important issues in light of their practical application to Sb(V)-containing wastewater treatments.

### 3.5. Properties of Precipitates Formed during Sb(V) Reduction by Strains AR-2 and AR-3

**Figure 3:**
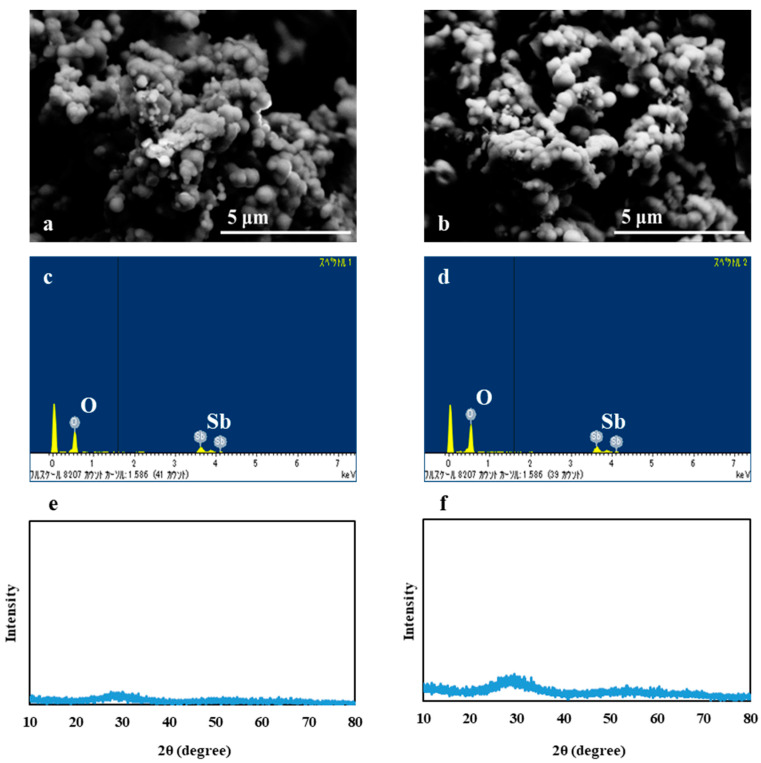
Scanning electron micrographs (**a**,**b**), energy-dispersive X-ray spectroscopy spectra (**c**,**d**), and X-ray diffraction patterns (**e**,**f**) of precipitates formed during anaerobic Sb(V) reduction for 7 d by strains AR-2 (**a**,**c**,**e**, respectively) and AR-3 (**b**,**d**,**f**, respectively).

The scanning electron micrographs of white precipitates formed during anaerobic Sb(V) respiration of strains AR-2 and AR-3 are shown in Figure 3a,b, respectively. The precipitates were produced extracellularly by both strains, and the aggregates of spherical particles measured 0.1–0.5 µm. EDX analysis showed that the particles produced by both strains comprised Sb and O (Figure 3c,d), with an atomic ratio (Sb:O) of approximately 1:4 (Table 2). The results suggested that the precipitates were likely solid forms of Sb_2_O_3_ and/or Sb(OH)_3_. Sb(OH)_3_ could be formed by the biological reduction of Sb(OH)_6_^−^ and further dehydrated to form Sb_2_O_3_ [16,28].

Earlier studies have reported that microbial Sb(V) reduction could form Sb_2_O_3_ precipitates as cubic (sénarmontite) and orthorhombic polymorphs (valentinite) or amorphous biominerals [16,27,29,30]. Nguyen et al. also reported the simultaneous formation of amorphous and crystallized Sb_2_O_3_ as products of microbial Sb(V) reduction [30]. In contrast, Nguyen and Lee demonstrated the formation of Sb(OH)_3_ precipitates as amorphous particles [17]. Based on the XRD patterns registered in the Powder Diffraction File (PDF) database of the International Center for Diffraction Data, strong peaks of crystalline Sb compounds appeared to be detected at 2θ of 27.698, 32.077, and 45.985 (cubic Sb_2_O_3_; PDF#05-0534) or of 25.172, 28.382, and 28.605 (orthorhombic Sb_2_O_3_; PDF#11-0689) in XRD analysis. However, XRD analysis of the precipitates formed during Sb(V) reduction by strains AR-2 and AR-3 did not detect any significant peaks (Figure 3e,f), which was possibly resulted from the very small particle size and/or amorphous structure. Based on the similarity in the size and structure, the precipitates formed by strains AR-2 and AR-3 in this study were likely amorphous Sb(OH)_3_ solids, as found by Nguyen and Lee [17]. Amorphous Sb(OH)_3_ could develop into crystallized Sb_2_O_3_ by a prolonged incubation time. Increased particle size and crystallization were observed when Sb(V) reduction experiments with strain AR-2 were extended to 35 d, whereas similar crystallization was not detected during Sb(V) reduction by strain AR-3 (Figure S7), which requires further study.

## 4. Conclusions

This study was the first to successfully isolate Sb(V)-reducing bacteria that are capable of reducing Sb(V) not only under strictly anaerobic conditions but also under microaerobic conditions. *Dechloromonas* sp. AR-2 and *Propionivibrio* sp. AR-3, whose Sb(V) reduction abilities were characterized in this study, would be useful in the practical treatment of Sb-containing wastewater. A more detailed understanding of Sb(V)-reducing abilities of the isolated strains from the viewpoint of practical applications, such as utilizable carbon sources (electron donors), utilization and influence of other electron acceptors (e.g., nitrate, sulfate and ferric ions) and other environmental parameters affecting their growth and Sb(V) reduction, is of great importance and currently under way. Further, the novel evidence that microbial Sb(V) reduction can occur even under microaerobic conditions would deepen our understanding of the fate of Sb compounds in the environment.

## Figures and Tables

**Table 1:** Physiological and phylogenetic characteristics of strains AR-2 and AR-3.

Strain	AR-2	AR-3
Cellular morphology	Rod (0.7–0.8 × 1.0–2.0 μm)	Rod (0.5–0.7 × 1.5–3.0 μm)
Gram stain	−	−
Spore	−	−
Motility	+	+
Catalase test	+	+
Oxidase test	+	+
O/F test	−	−
Growable temperature in LB medium	15–37 °C	15–37 °C
Growable pH in LB medium	6–8.5	5–8.5
Closest relative of 16S rRNA gene sequence	*Dechloromonas agitata* CKB^T^ (Similarity: 98.4%)	*Propionivibrio militaris* MP^T^ (Similarity: 99.8%)

+: Positive; −: Negative; O/F test: oxidation-fermentation test.

**Table 2:** Elemental distribution in precipitates formed through anaerobic Sb(V) reduction by strains AR-2 and AR-3.

Strain	Elemental Distribution in Precipitates (%)		
	O	S	Sb
AR-2	78.2	nd	21.8
AR-3	82.4	0.1	17.5

nd, not detected.

## Supplementary Materials

The followings are available online at https://www.mdpi.com/2076-2607/8/9/1435/s1, Table S1. Composition of the inorganic solution, Table S2. Buffers used to adjust the pH of LB broth in experiments to test the growth ability of strains AR-2 and AR-3, Table S3. Results of biochemical and assimilation tests of strains AR-2 and AR-3, Figure S1. Relationships between cell concentrations and OD_600_ values (a and b) or protein concentrations (c and d) for strains AR-2 (a and c, respectively) and AR-3 (b and d, respectively), Figure S2. Appearance of the enrichment culture before the onset of enrichment (a) and after the 1st (b) and 6th batches (c), Figure S3. Acetate consumption during anaerobic Sb(V) reduction by strains AR-2 and AR-3. Error bars indicate the standard deviation (*n* = 3), Figure S4. Precipitates formed during Sb(V) reduction experiments under microaerobic (a) and anaerobic conditions (b), Figure S5. Positive correlations between cell growth represented by the increase in protein concentration and acetate consumption (ΔAcetate) (a and b) and between cell growth and Sb(V) decline (ΔSb(V)) (c and d) during anaerobic Sb(V) reduction by strains AR-2 (a and c, respectively) and AR-3 (b and d, respectively), Figure S6. Acetate consumption during anaerobic, microaerobic and aerobic Sb(V) reduction experiments by strains AR-2 (a) and AR-3 (b). Error bars indicate the standard deviation (*n* = 3), Figure S7. Scanning electron micrographs of precipitates after 35 d in anaerobic Sb(V) reduction experiments using strains AR-2 (a) and AR-3 (b).
