# Supplementary material for: Isolation and Characterization of Facultative-Anaerobic Antimonate-Reducing Bacteria
Source: Microorganisms. 2020 Sep 18;8(9):1435. doi: 10.3390/microorganisms8091435 (PMC7563848; doi:10.3390/microorganisms8091435)
Supplement: Supplementary file 1 [file microorganisms-08-01435-s001.pdf]

Table S1. Composition of the inorganic solution

Component	Concentration
NaCl	1.2 g/L
KCl	0.3 g/L
NH_4Cl	0.3 g/L
KH_2PO_4	0.2 g/L
Na_2SO_4	0.009 g/L
$MgCl_2 \cdot 6H_2O$	0.4 g/L
HEPES (pH 7.0)	20 mM

Table S2. Buffers used to adjust the pH of LB broth in experiments to test the growth ability of strains AR-2 and AR-3

pH	Buffer
5.0	MES (2-(*N*-Morpholinoethanesulfonic acid)
6.0	MES
7.0	HEPES (2-[4-(2-Hydroxyethyl)-1-piperazinyl]ethanesulfonic acid)
7.8	CHES (*N*-Cyclohexyl-2-aminoethanesulfonic acid)
8.5	CAPS (*N*-Cyclohexyl-3-aminopropanesulfonic acid)

Table S3. Results of biochemical and assimilation tests of strains AR-2 and AR-3

Reaction/Enzyme		AR-2	AR-3
Biochemical tests	Nitrate reduction	+	+
	Indole production	-	-
	Glucose fermentation	-	-
	Arginine dihydrolase	-	-
	Urease	-	-
	Hydrolysis (esculin)	-	-
	Hydrolysis (gelatin)	-	-
	β-Galactosidase	-	-
	Cytochrome oxidase	+	+
Assimilation tests	Glucose	-	-
	L-Arabinose	-	-
	D-Mannose	-	-
	D-Mannitol	-	-
	N-acetyl-D-glucosamine	-	-
	Maltose	+	+
	Potassium gluconate	-	-
	n-Capric acid	-	-
	Adipic acid	-	-
	DL-Hydroxybutanedioic acid	-	-
	Sodium citrate	-	-
	Phenyl acetate	+	-

+: Positive, -: Negative

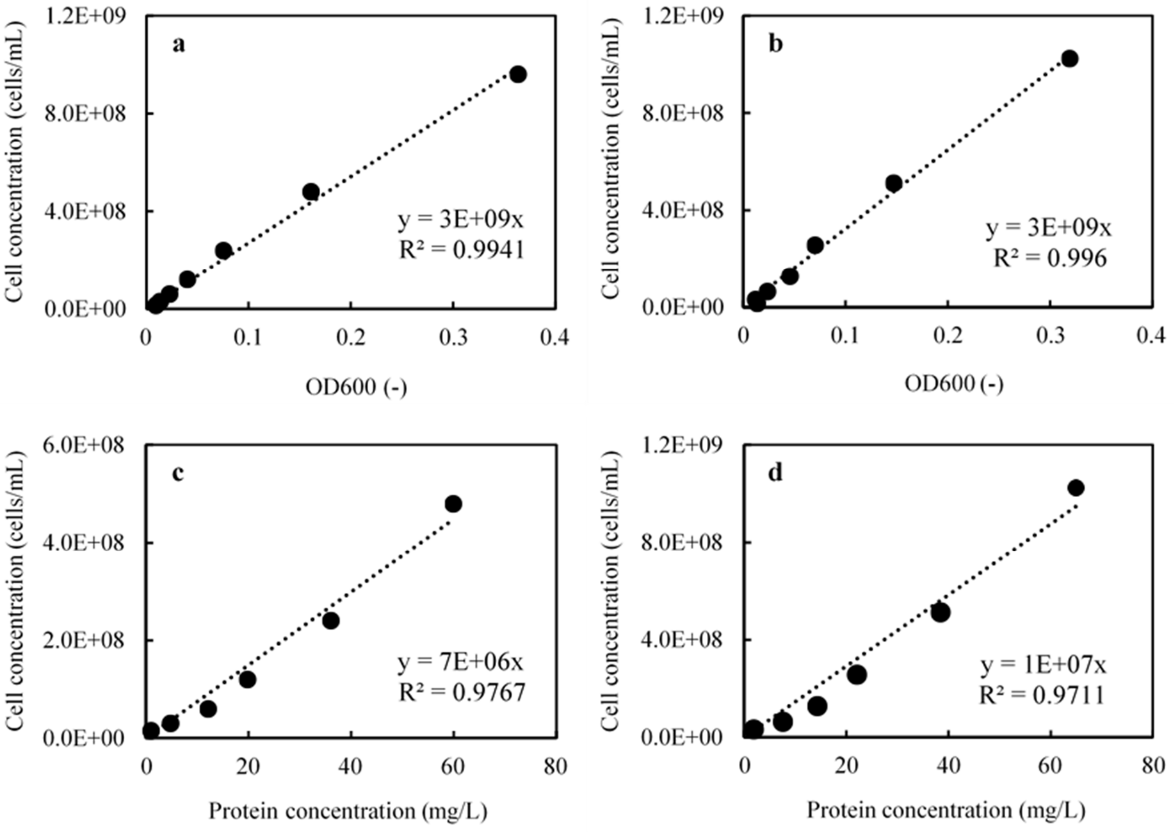

Figure S1. Relationships between cell concentrations and OD_{600} values (a and b) or protein concentrations (c and d) for strains AR-2 (a and c, respectively) and AR-3 (b and d, respectively).

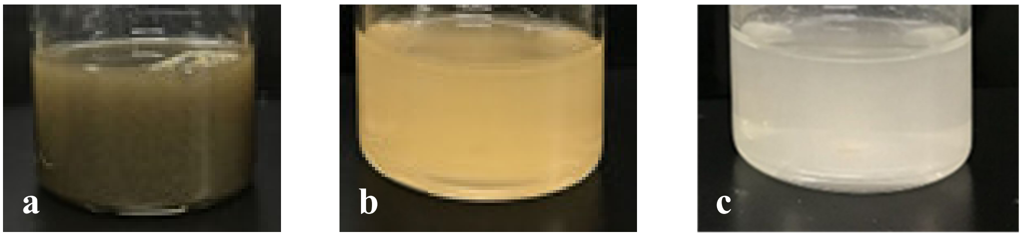

Figure S2. Appearance of the enrichment culture before the onset of enrichment (a) and after the 1st (b) and 6th batches (c).

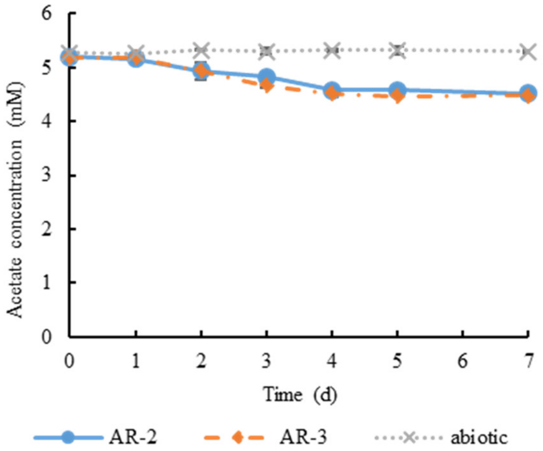

Figure S3. Acetate consumption during anaerobic Sb(V) reduction by strains AR-2 and AR-3. Error bars indicate the standard deviation (n = 3).

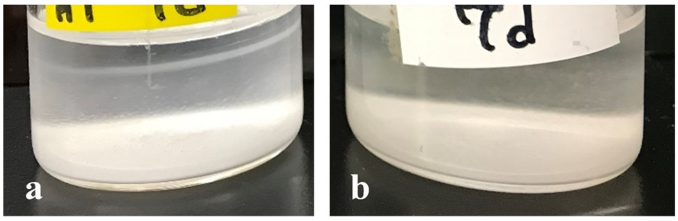

Figure S4. Precipitates formed during Sb(V) reduction experiments under microaerobic (a) and anaerobic conditions (b).

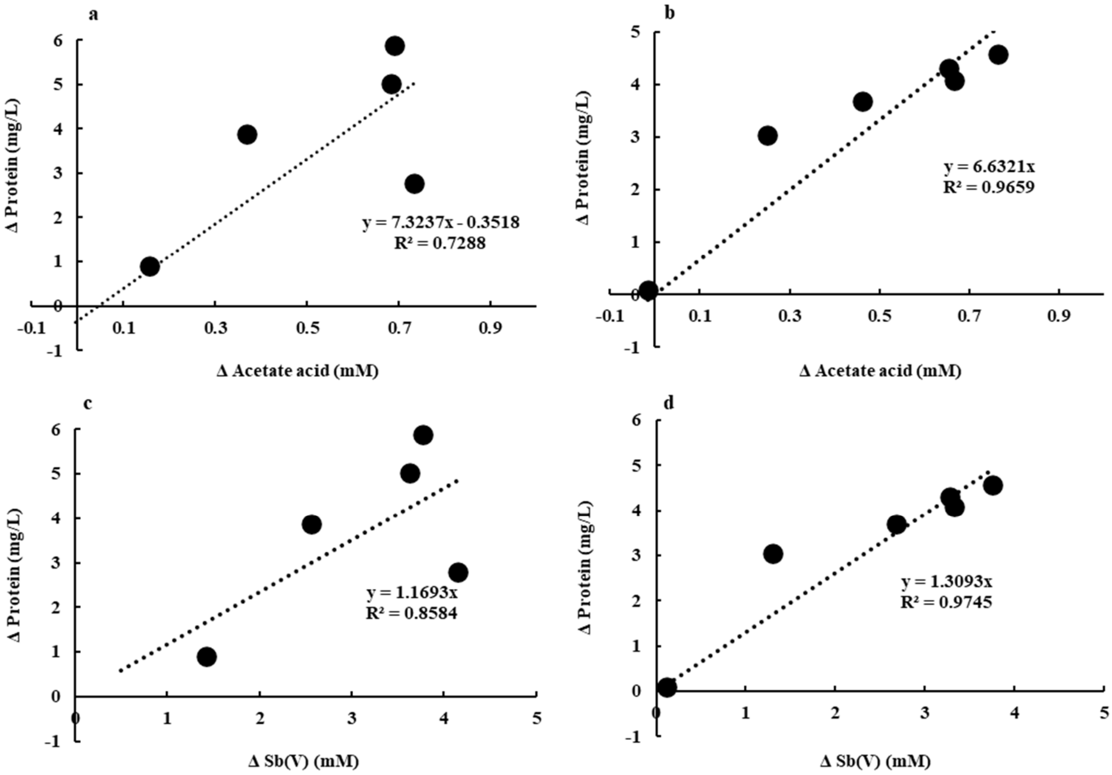

Figure S5. Positive correlations between cell growth represented by the increase of protein concentration and acetate consumption (ΔAcetate) (a and b) and between cell growth and Sb(V) decline (ΔSb(V)) (c and d) during anaerobic Sb(V) reduction by strains AR-2 (a and c, respectively) and AR-3 (b and d, respectively).

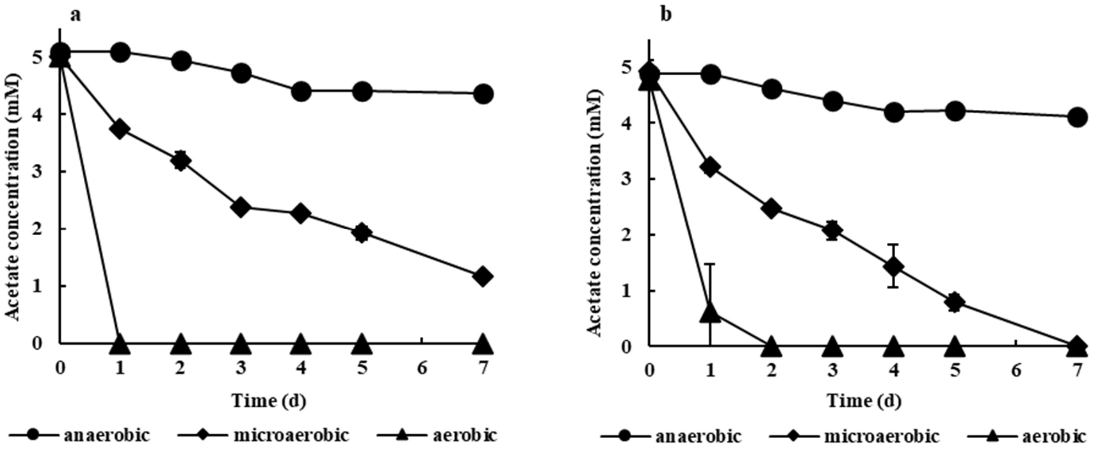

Figure S6. Acetate consumption during anaerobic, microaerobic and aerobic Sb(V) reduction experiments by strains AR-2 (a) and AR-3 (b). Error bars indicate the standard deviation (n = 3).

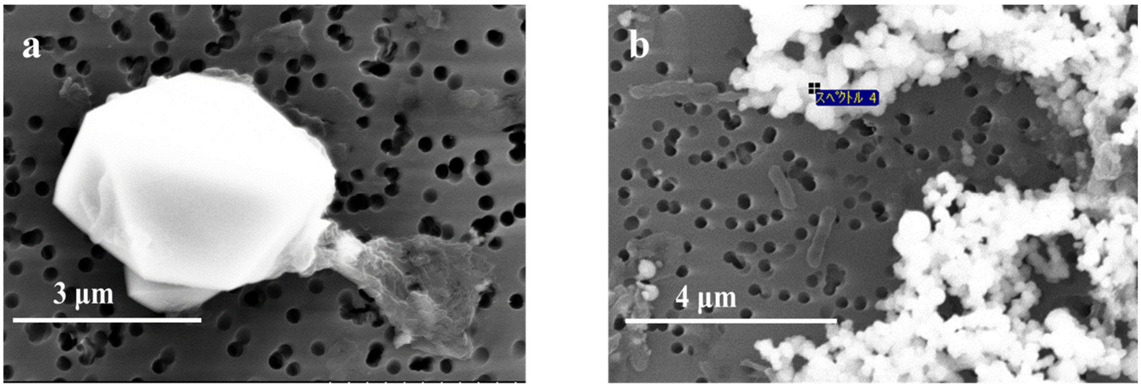

Figure S7. Scanning electron micrographs of precipitates after 35 d in anaerobic Sb(V) reduction experiments using strains AR-2 (a) and AR-3 (b).
